# Case report: bipolar disorder as the first manifestation of CADASIL

**DOI:** 10.1186/1471-244X-14-175

**Published:** 2014-06-14

**Authors:** Soyeon Park, Boram Park, Min Kyung Koh, Yeon Ho Joo

**Affiliations:** 1 Department of Psychiatry, University of Ulsan College of Medicine, Asan Medical Center, 88 Olympic-ro 43-gil, Songpa-gu, Seoul 136-736, Korea; 2 Department of Psychology, Asan Medical Center, 88 Olympic-ro 43-gil, Songpa-gu, Seoul 136-736, South Korea

**Keywords:** CADASIL, Bipolar disorder, Mood disorder, NOTCH3

## Abstract

**Background:**

Cerebral autosomal dominant arteriopathy with subcortical infarcts and leukoencephalopathy (CADASIL) is an inherited cerebrovascular disease, clinically characterized by variable manifestations of migraine, recurrent transient ischemic attack or lacunar strokes, cognitive decline, and mood disturbances. However, manic episodes have rarely been documented as an initial symptom of CADASIL and bipolar disorder presenting as the first manifestation in CADASIL has not been reported previously from evaluations by psychiatrists or psychological testing by psychologists.

**Case presentation:**

A 53 year old woman developed symptoms of mania in her 50s leading to a personality change involving a continuously labile mood and irritability over a number of years. Neuropsychological testing revealed an intact memory, but impairment in attention and executive function. In the Rorschach test, she showed a high level of cognitive rigidity. Magnetic resonance imaging findings were very consistent with a diagnosis of CADASIL, which was confirmed by genetic testing for NOTCH3 mutations. Atypical antipsychotics proved to be helpful in treating her manic symptoms and for behavior control.

**Conclusion:**

We present a novel case of CADASIL that first presented as bipolar disorder. We contend that when patients show a late onset personality change or chronically irritable mood that deteriorates over many years, an organic cause such as CADASIL must be considered. Further studies are needed to better understand the exact impacts of cerebral tissue lesions and psychiatric symptoms in CADASIL patients.

## Background

The genetic contributions to bipolar disorder (BD) have been well characterized, with an increased risk, estimated to be 8-fold higher than the prevalence in the general population, in siblings with affected probands [1]. It is believed that BD is a complex genetic disease that is caused by various interactions between the environment and by multiple genes [1]. Although a high degree of heritability is known for BD, the mode of inheritance for this disorder appears complex, indicating that it is non-Mendelian in nature and involves genes that have yet to be identified [2]. One of the suggestions for why BD has not been clarified as a single genetic disease is heterogeneity. Research on BD subtypes is still at an early stage, and the concepts being developed to explain this disease are changing continuously. Recently, the concept of a bipolar spectrum disorder was introduced to try to understand BD from a continuous line into the past. Hence, there have been attempts to distinguish between BD subtypes to better understand the etiology of this disorder.

Cerebral autosomal dominant arteriopathy with subcortical infarcts and leukoencephalopathy (CADASIL) is a rare hereditary disease caused by mutations in the NOTCH3 gene on chromosome 19p13.1 [3]. CADASIL is a systemic angiopathy and it is characterized by recurrent transient ischemic attacks (TIA), strokes, vascular dementia, mood disturbances and motor disability. If CADASIL arises at an age below 20, the most common first symptom is migraine. However, if the first symptoms of CADASIL occur in persons over 50, they can rapidly progress to a more serious condition such as depression [4]. Because these cases do not show neurological abnormalities, they are commonly diagnosed as a mood disorder only. The diagnosis of CADASIL is therefore often delayed or missed in psychiatric clinics.

Although there has been considerable neurological research on CADASIL, there have been fewer studies of the mood disorders associated with this disease. Psychiatric disturbances have been mentioned by almost all previous studies of CADASIL, but most of these reports have only addressed the presence of psychiatric disorders among the symptoms of the disease and have not focused specifically on the psychiatric aspects of CADASIL [5]. Hence, prior CADASIL studies have not incorporated a psychiatric evaluation or provided any detailed descriptions of psychiatric symptoms. In our current report, we present a case of CADASIL that first presented as BD. Through our analysis of this case, we provide new insights into the issues around the initial differential diagnosis of CADASIL and also the appropriate interventions for these patients.

## Case presentation

A 53-year-old woman visited our out-patient psychiatric clinic in March 2013. Her daughter reported that she had become excessively talkative, impulsive, and verbally aggressive. The changes in her personality had begun to appear in 2008 and her symptoms of hostility had become more severe recently. Her premorbid personality was described as reserved, diligent, frugal, and calm and she had worked in a market for more than 20 years. However, during the previous 5 years, she had begun to speak garrulously and often lost the thread of a conversation and included many irrelevant details when providing an explanation. She had become much more easily irritable and angry, which took the form of yelling and throwing objects. She had also begun to argue over seemingly unimportant matters with her neighbors. She had also begun to impulse buy multiple items also make many unnecessary purchases for her family. She had also become very hyperactive in her work life and social life. She did not display any sleep or eating disorders at the time of her visit.

The patient’s husband had an explosive temperament and alcohol problems which were a source of great stress and concern for her. Since 2000, she had begun to irregularly attend a local psychiatric clinic to receive medications for a depressed mood and insomnia. At the time of these visits, she was taking both antidepressants and sleeping pills. However, she and her family denied that she had experienced any depressive episodes or had ever been diagnosed with a mood disorder. She had no history of head trauma but had hypertension and diabetes, both of which are cardiovascular risk factors. There was also no history of migraine, a prior TIA, or stroke reported by the patient.

Her family history further revealed that her father had died at 76 years of age after having suffered a stroke and that he had hypertension, diabetes and a history of depression including a suicide attempt. However, her mother is 76 years old and in good health. She has three younger brothers, the oldest of whom is 51 years old and has been diagnosed with cerebellar atrophy. He also has dysarthira and cerebellar ataxia. Those symptoms had gradually developed within the previous 2 years. Her second brother had suffered from depression and had committed suicide 20 years previously. Her youngest brother had no health issues, but his 17-year-old son had been diagnosed with epilepsy. She also has a son and a daughter. Her son suffers from migraine attacks but her daughter is healthy.

When her mental condition was assessed she showed no evidence of delusions or hallucinations, but she did have pressured speech, circumstantiality, labile moods, poor impulse control, and made grandiose plans. She demonstrated no insights into the changes in her personality and behavior. Bedside neuropsychological testing revealed total scores that were within the normal range (K-MMSE 29/30). However, a Montreal Cognitive Assessment test (MOCA-K 25/30) revealed a failure to perform the trail making test (TMT) and copy cube. In the attention subtest, she tapped on the wrong letters and failed to correct this. In the language subtest, she could speak more than 11 words in 60 seconds but the all of the words began with the same sound with a different suffix, for example, like, liked, likely, look, looked, looking. In a delayed recall subtest, she recalled only two words correctly out of five. She was successful with Clock drawing, the Luria’s fist-edge-palm test and the loop test.

Due to a suspected late-onset bipolar disorder with chronic manic features in this case, neuropsychological testing was performed by a clinical psychologist. To evaluate this patient’s comprehensive cognitive function, we performed the following tests: BGT, Clock Drawing Test, Color Trail Test (CTT) 1/2, Korean Auditory Verbal Learning Test (K-AVLT), Stroop Test, Word/Figural Fluency Test, Executive Complex Figure Test, Grooved Pegboard Test, Finger Tapping Test, Hand Dynamometer, MMPI-II, Sentence Completion Test (SCT), Wisconsin Card Sorting Test (WCST) and Rorschach Test.

The K-AVLT findings showed that the patient had a normal ability to encode and retrieve verbal information. However her abilities in terms of tracking sequential stimulations, dividing her attention and inhibitory control function were found to be severely impaired, with a poor score in terms of attention and executive function in the CTT tests (<1% percentile). Cognitive productivity was also poor as reflected in impaired word/figure fluency. In the WCST, the patient showed 18 perseverative responses (50% percentile) and 47 non-perseverative responses (<1% percentile). Her potential for perseveration was therefore low, and she showed an impaired efficient sorting tendency. The results of the Rorschach test indicated a strong cognitive rigidity in this patient as she only responded to the same content repetitively. Finally, the results of the MMPI-II showed that scales 1 and 9 were all higher than 65 T, indicating that this patient was complaining of physical pain or discomfort and making efforts to cope with difficulties in her cognitive function.

In conclusion, neuropsychological testing of our current case revealed an intact memory, but impairment in both attention and executive function. Although the patient’s perseveration tendency was not clear, she revealed a high level of cognitive rigidity in the Rorschach test. The results of the neurological examination of this case were unremarkable and other systemic examinations showed no abnormalities. The results of routine laboratory tests including VDRL, HIV antibodies and thyroid function were also unremarkable apart from hyperlipidemia. MRI analysis of the brain was performed and revealed severe leukoencephalopathy. There was a high signal intensity found by T2 weighted images (T2W1) and low signal intensity in the left pons. In fluid-attenuated inversion-recovery (FLAIR) images, a high signal intensity was prominent on the periventricular white matter, both basal ganglia, thalamus, and the external capsule. These magnetic resonance imaging (MRI) findings are very consistent with a diagnosis of CADASIL. Since the clinical and MRI findings for this patient were highly suggestive of CADASIL, genetic testing for NOTCH3 mutations was performed. As expected, a heterogeneous mutation was detected in this patient that causes an arginine to proline substitution in exon 3 of chromosome 19 p13.2-13.1 (c.224G > C).

During her hospital treatment, her manic symptoms, including a labile mood, talkativeness and impulsivity, partially responded to atypical antipsychotics (quetiapine, 800 mg) and benzodiazepine (bromazepam, 6 mg) treatment. She was also prescribed with amlodipine (novasc), metformin (diabex), and atorvastatin (lipitor) for the management of the vascular risk factors, hypertension, diabetes, and hyperlipidemia, respectively. She was able to be discharged after two weeks in hospital. At the time of discharge, she was no longer hostile or aggressive but still showed slight impulsivity and mood instability symptoms. Her initial Montgomery-Asberg Depression Scale (MADRS) and Young Mania Rating Scale (YMRS) scores were 24 and 37, respectively. Two weeks after treatment, these values were lowered to 17 and 25, respectively. Her Clinical Global Impression-Severity Scale (CGI-S) score was 6 (severely ill) at initial presentation and 4 (moderately ill) at hospital discharge. Her Global Assessment of Functioning (GAF) score also slightly improved from 50 to 60 after treatment.

## Conclusions

CADASIL is an example of pleiotropism i.e. single gene effects on multiple phenotypic traits. CASASIL is a monogenic disease caused by a dysfunction in the NOTCH3 gene localized on chromosome 19p13.1, but shows a wide variety of clinical symptoms. This disease is characterized by five main symptoms: migraine with aura, subcortical ischemic events, mood disturbance, apathy, and cognitive impairment [6]. Psychiatric symptoms, mainly episodes of mood disturbances, are seen in 23% of CADASIL cases [5].

Our current case of a bipolar clinical presentation of CADASIL without remarkable neurologic deficits provides some important new insights into this disorder. Our patient was interviewed and described by a psychiatrist. Psychiatric disturbances in patients with CADASIL are usually reported to occur during the course of the disease [5], and are often the first recorded symptoms because the affected patients will first be admitted to a psychiatric clinics. The initial symptoms of CADASIL vary with the age of onset, but migraine and TIA are the most commonly reported initial symptoms [4]. Depression is the second most common first symptom in patients who first present with CADASIL at an age of over 50 [4]. However, manic episodes have rarely been documented as an initial symptom of CADASIL [4,7]. A recent CADASIL review article, has highlighted that personality and behavioral disorders, such as irritability and aggressiveness, were reported in only 7 cases (2%) of a previous 454 patient cohort and that BD was documented in 9 cases (2%) of a 451 patient series [5]. However, these studies were limited by the fact that clinical manifestations were mainly defined by medical specialists or medical records and not through psychiatric evaluations. Also, manic symptoms appear to be uncommon in CADASIL patients and may therefore have generated less interest among clinicians.

Few studies have investigated mood disorder with a more specific focus on psychiatric disorders in CADASIL. Layhe et al. [8] reported two CADASIL cases who had been admitted to a gerontopsychiatric hospital with psychopathological manifestations at the onset of the disorder. One of these patients had experienced a submanic episode who had become impulsive, verbally aggressive, irritable, and sexually preoccupied. Taylor et al. [9] have described a male CADASIL patient who showed persistent behavioral disturbances, irritability, and angry outbursts that lasted for 4years. Gamakaranage et al. [10] have also described a CADASIL case with a changed personality involving mood swings, aggressive behavior, and cognitive deficit [10]. Although this patient seemed to show symptoms of mania, this was considered to be just a personality problem as the causes were attributed to the persistence of manic symptoms for many years. This patient was not interviewed by psychiatrist. Only one previous report has described a case of CADASIL with mania [11]. However, it had not included detailed descriptions of neuropsycholocial evaluations or psychiatric treatment.

In our present case report, the symptoms of BD which had begun when our patient was aged in her 50s seemed for many years to be a personality change due to a continuously labile mood and irritability. She had specific findings on a cerebral MRI and did not respond well to medication. She showed an intact memory, but neuropsychological testing revealed impairment of both attention and executive function. However, detailed descriptions of psychiatric symptoms, neuropsychological testing and treatments are available for this patient. We propose several clinical characteristics of CADASIL presenting as BD. All CADASIL cases for which manic symptoms have been reported including our current case have been documented with a labile mood, irritability, and verbal aggression, with no euphoria or elated mood. All CADASIL patients show a first manifestation of late-onset disease after reaching the age of 50. In addition, as they show a chronic symptom course which has progressed for years, they have often been diagnosed with a personality problem. We contend therefore that when patients show a late onset personality change, and a chronically labile or irritable mood that deteriorates over many years, an organic cause like CADASIL must be considered.

The most prominent neurologic deficits reported in CADASIL patients to date manifest as a reduced processing speed as assessed, for example, by the timed measurements in the TMT and the symbol digit tests. Indeed, abnormalities that are revealed by the TMT B, which measures processing speeds and set shifting, are remarkably consistent across all published studies on CADASIL including our current case [12]. Cognitive decline predominantly affects frontal lobe functions and is characterized by impaired executive function and poor concentration. Cognitive decline may also commence insidiously before the onset of symptomatic ischemic episodes in CADASIL [13]. These impairments are suggestive of dysfunction in the subcortical-frontal network [12,14]. Memory and other cognitive functions are relatively preserved at the early stages of CADASIL [12].

Patients presenting with psychiatric symptoms should undergo a brain MRI if they have a history of headache, stroke-like episodes, or cognitive changes and a positive family history of these manifestations [15]. Hyperintensities of the external capsule and the anterior pole of the temporal lobe are highly suggestive of CADASIL [16]. Hyperintense lesions have been found in both subcortical white matter and gray nuclei and occur more frequently in BD subjects than in age-matched controls [17]. Abnormalities converge in the prefrontal white matter and, in particular, in the tracts that connect prefrontal regions and subcortical gray matter structures known to be involved in emotion [17]. It is noteworthy that the MRI changes previously known in BD are strikingly similar to those reported in CADASIL. It might be postulated that psychiatric manifestations in CADASIL depend on the extent of the damage the cortical-subcortical circuits, as evidenced by white matter lesions and lacunar infarcts [18]. Recently, ischemic subcortical lesions are reputed to be important risk factors for the onset of mood disorders [19]. However, information is still lacking regarding the relationship between mood disorders and lesions detected on MRI. The NOTCH pathways have been postulated as a cause of white matter changes in BD [17].

**Figure 1:**
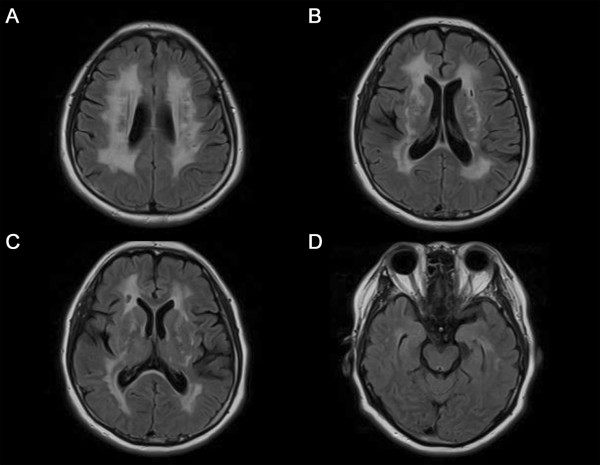
Cerebral MRI with fluid-attenuated inversion-recovery (FLAIR) showing high-signal intensity lesions in the periventricular white matter, both basal ganglia, thalamus, and external capsule (A-C), but not prominent in the anterior temporal poles (D).

Although some studies of CADASIL have described phenotype-genotype correlations, the genotype cannot be used to predict the phenotype in this disease [20]. The R75P mutation identified in our current case is a novel mutation in a CADASIL patient but is relatively common in Koreans [21]. In addition, this mutation is related to arginine, whereas previous CADASIL case reports have frequently described the involvement of a cysteine residue. However, several mutations at arginine residues in EGF-like repeats have been identified previously in other proteins that are functionally related to NOTCH3 [21]. Whilst anterior temporal hyperintensities have been commonly observed in 75% of CADASIL cases without an R75P mutation, they have not been observed in any patients with this mutation [21]. Consistently, our current patient did not show prominent anterior temporal hyperintensities (Figure 1D).

However, this study was retrospective and has some limitations. This was not confirmed GOM by a skin biopsy, and we investigated exon 2–11, 18 of NOTCH3 which was known as mutation of CASASIL. Because we did not survey other exons from 12 to 15 or 20–23 have been reported [22] expressing NOTCH3 and exclude the possibility of other mutations related to cysteine.

CADASIL is likely to be an underdiagnosed disease, particularly in psychiatric patients. It is therefore important to highlight CADASIL to psychiatrists as a potential differential diagnosis. When BD patients show different clinical features such as resistance to antipsychotics, and late onset and chronic progression, the neurological signs and detection of white-matter MRI abnormalities, as well as a positive family history of stroke, are helpful for diagnosing CADASIL [23]. Testing which is particularly sensitive for the detection of the early cognitive changes can then be performed such as digit span back-forwards, TMT-B, Stroop, and WCST tests. These tests have revealed that patients with CADASIL show preservation of encoding processes even though memory retrieval is impaired [23].

There is currently no treatment with a proven efficacy for CADASIL. Because CADASIL is a vascular disorder responsible for cerebral ischemic events, treatment of vascular risk factors is needed for secondary prevention in affected patients. Hypertension, hypercholesterolemia and diabetes should also be treated very strictly in these cases. Donepezil can be prescribed for improving executive function, and other symptoms should be treated symptomatically in CADASIL patients [24]. Yuan et al., have reported that valproate (VPA), an anticonvulsant and mood stabilizer activates the NOTCH3/c-FLIP cascade, conferring cytoprotective effects on vascular smooth muscle cells [25]. Thus, VPA may be useful in the treatment of CADASIL, particularly in patients showing BD. In our current case, atypical antipsychotics proved to be helpful in behavior control. Further studies are needed to better understand the exact impacts of cerebral tissue lesions and psychiatric symptoms in CADASIL patients.

### Consent

Written informed consent was obtained from the patient for publication of this case report and any accompanying images.

## Competing interests

All authors disclose no potential conflicts of interest include employment, consultancies, stock ownership, honoraria, paid expert testimony, patient applications/registrations, and grants or other funding.

## Authors’ contributions

S Park participated in the design of the study and drafted the manuscript. BR Park made acquisition and interpretation of a psychiatric history of a case report part and helped to draft the manuscript. MKK made acquisition and interpretation of psychological tests of a case report part and helped to draft the manuscript. YHJ conceived of the study, and participated in its design and coordination and helped to draft the manuscript. Each author has participated sufficiently in the work to take public responsibility for appropriate portions of the content. All authors read and approved the final manuscript.

## Pre-publication history

The pre-publication history for this paper can be accessed here:

http://www.biomedcentral.com/1471-244X/14/175/prepub
